# Clinical and microbial correlates of response to lifestyle intervention in pediatric metabolic dysfunction-associated steatotic liver disease

**DOI:** 10.1186/s13099-026-00798-5

**Published:** 2026-01-18

**Authors:** Jong Woo Hahn, Jin Gyu Lim, Kyung Jae Lee, Jin Soo Moon, Tae Hyeong Kim, Yejun Son, Dong Keon Yon, Yun Jung Lee, Yuri Seo, Jihyun Park, Seunghyun Lee, Donghyun Kim, Jae Sung Ko

**Affiliations:** 1 Department of Pediatrics, Seoul National University College of Medicine, Seoul, South Korea; 2 Department of Pediatrics, Seoul National University Bundang Hospital, Seongnam, Gyeonggi-do South Korea; 3 Department of Pediatrics, Kyung Hee University Hospital at Gangdong, Seoul, Korea; 4 Center for Digital Health, Medical Science Research Institute, Kyung Hee University College of Medicine, Seoul, South Korea; 5 Department of Pediatrics, Kyung Hee University Medical Center, Kyung Hee University College of Medicine, Seoul, South Korea; 6 Department of Food Service and Nutrition Care, Seoul National University Hospital, Seoul, South Korea; 7 Department of Biomedical Sciences, Seoul National University College of Medicine, Seoul, South Korea; 8 Department of Radiology, Seoul National University College of Medicine, Seoul, South Korea; 9 Department of Microbiology and Immunology, Seoul National University College of Medicine, Seoul, South Korea; 10 Seoul National University College of Medicine, Seoul National University Children’s Hospital, 101 Daehak-ro, Jongno-Gu, Seoul, 03080 Korea

**Keywords:** Fatty liver, Lifestyle, Pediatric obesity, Gut microbiota

## Abstract

**Background:**

Lifestyle modifications are fundamental in managing metabolic dysfunction-associated steatotic liver disease (MASLD). This study aimed to evaluate the effects of a 12-week lifestyle intervention on hepatic steatosis, liver function, and gut microbiota composition in pediatric MASLD patients, and to explore clinical and baseline microbial features associated with treatment response.

**Results:**

A total of 40 patients were recruited, and 31 were included after applying exclusion criteria. After 12 weeks, significant reductions were observed in body weight, waist circumference, liver enzymes, and triglycerides. MRI-measured hepatic steatosis decreased from 27.1% to 20.8% (*p* < 0.05). Greater reductions in hepatic fat were associated with higher daily step counts and a higher proportion of dietary protein intake. Baseline gut microbial composition differed between clinical responder groups. *Clostridium sensu stricto 1* was enriched in participants with significant weight loss, *Faecalibacterium* in those with ALT or GGT improvement, and *Lachnospiraceae_ND3007_group* in those with MRI-PDFF reduction, with baseline microbial profiles discriminating responders with AUC ≥ 0.75.

**Conclusions:**

A 12-week lifestyle intervention led to significant improvements in hepatic steatosis, metabolic parameters, and anthropometric measures in pediatric MASLD. Baseline gut microbial profiles differed between individuals with metabolic improvements, suggesting a potential association between pre-intervention microbiome composition and treatment response.

**Trial registration:**

The Clinical Research Information Service of the Korea Center for Disease Control and Prevention, Number: KCT0010340.

**Supplementary Information:**

The online version contains supplementary material available at 10.1186/s13099-026-00798-5.

## Introduction

Metabolic dysfunction-associated steatotic liver disease (MASLD) is diagnosed when there is an accumulation of liver fat by 5% or more, along with meeting at least one cardiometabolic criterion, and in the absence of other identifiable causes of steatosis, such as alcohol consumption [1]. While liver biopsy has traditionally been considered the gold standard for diagnosing MASLD, a meta-analysis study conducted in the UK has evaluated that magnetic resonance imaging (MRI) can also diagnose MASLD and assess its severity [2]. The prevalence of MASLD has been steadily increasing worldwide. Recent global estimates indicate a prevalence of approximately 10–20% among children and adolescents, and up to 40–50% among those with obesity, with higher rates reported in Asian populations [3]. High-calorie diets, excessive fat intake, and Western dietary patterns characterized by refined carbohydrates, added sugar (particularly fructose), and physical inactivity are known major contributors to the development and progression of MASLD [4]. 

Changes in gut microbiota have also been reported in MASLD patients, associated with dietary shifts to high-fat diets, obesity, reduced microbial gene diversity, decreased resistance to oxidative stress, and increased insulin resistance [5, 6]. Numerous studies have analyzed gut microbiota in MASLD and attempted to alter its composition through interventions. Clinical trials involving synbiotics, dietary modifications, and exercise have shown that such interventions can increase microbial diversity and enrich beneficial taxa such as *Bacteroidetes*, *Faecalibacterium*, and *Parabacteroides*, while reducing potentially pathogenic groups including *Actinobacteria* and *Oscillibacte*r [7–10]. 

Given the rising incidence of pediatric obesity and MASLD, lifestyle modification through structured dietary and physical activity interventions is widely regarded as the cornerstone of treatment [4]. The HEALKIDS study was designed to evaluate the effects of a 12-week lifestyle intervention on hepatic steatosis, metabolic parameters, and gut microbiota composition in pediatric MASLD patients. The study further aims to identify potential non-invasive biomarkers associated with clinical improvement, thereby contributing to the development of targeted, individualized strategies for managing MASLD in children.

## Methods

### Study design

HEALKIDS, a prospective, single–arm interventional study, evaluated changes in liver fat, liver function, and the gut microbiome over 12–week lifestyle modifications, including diet and exercise. The trial was registered with the Clinical Research Information Service of the Korea Center for Disease Control and Prevention (KCT0010340). From October 2022 to June 2023, pediatric patients (aged 10–18) visiting Seoul National University Children’s Hospital with hepatic steatosis on liver MRI and at least one cardiometabolic criterion were included [1]. Exclusion criteria included other liver diseases, antibiotics use within the past 3 months, or other factors deemed inappropriate for participation. The study protocol was presented in Supplement material. All authors had access to the study data and reviewed and approved the final manuscript.

### Study intervention

Participants engaged in a 12-week lifestyle program that combined home-based exercise and individualized dietary counseling. The exercise component incorporated circuit and challenge training videos developed by Seoul Metropolitan Dongbu Hospital as part of the *Kkum-Namu (Dream Tree) Health Promotion Project*. Participants were encouraged to follow the weekly videos under parental supervision but were not required to adhere strictly to them. Instead, they were allowed to perform other equivalent moderate-to-high intensity aerobic physical activities for at least one hour per session, five times per week, aiming for 10,000 daily steps monitored via wearable devices. Participants completed the International Physical Activity Questionnaire (IPAQ) weekly, recording exercise duration, walking, and sedentary time, and submitted reports during outpatient visits. All participants received individualized dietary counseling by a nutritionist at baseline and were instructed to complete a 3-day food diary before each follow-up visit at weeks 4, 8, and 12. The education emphasized reduction of total caloric intake—particularly sugar and fat—while maintaining balanced macronutrient intake.

Compliance was assessed through pedometer-recorded step counts and MET-based physical-activity questionnaires for exercise, as well as 3-day food diaries evaluating total average daily intake and carbohydrate/protein/fat ratios for diet.

### Clinical assessments

At the beginning and end of the study, Z scores for weight, height, BMI, blood pressure, and waist circumference were calculated based on sex and age. Blood tests measured alanine aminotransferase (ALT), aspartate aminotransferase (AST), alkaline phosphatase (ALP), and γ-glutamyl transferase (GGT), as well as fasting glucose, fasting insulin, total cholesterol, high-density lipoprotein (HDL)–cholesterol, low-density lipoprotein (LDL)–cholesterol, triglycerides, and HbA1c. Participants showing a ≥ 10% relative reduction in ALT or GGT from baseline to week 12 were classified as having improved biochemical response. This exploratory threshold was chosen considering typical within-subject biological and analytical variability of liver enzymes and to minimize misclassification from minor fluctuations. At the beginning and end of the study, liver MRI assessed steatosis and fibrosis severity. Steatosis was graded as follows: grade 0 (healthy, < 6.4%), grade 1 (mild, 6.4%−17.4%), grade 2 (moderate, 17.4%−22.1%), and grade 3 (severe, > 22.1%) [11]. Fibrosis was classified as F0 (none, < 2.5 kPa), F1 (mild, 2.5–3.0 kPa), F2 (moderate, 3.0–3.5 kPa), F3 (severe, 3.5–4.0 kPa), and F4 (cirrhosis, 4.0–4.5 kPa) [12]. Hepatic steatosis improvement was defined as a ≥ 30% relative reduction in MRI-PDFF from baseline to week 12, in accordance with previous validation studies showing that this threshold predicts histologic improvement and fibrosis regression in nonalcoholic steatohepatitis [13, 14]. 

### Fecal microbiota analysis

Stool samples (5 g each) were collected before and after the 12-week exercise intervention, divided into at least three aliquots, and stored at −70 °C. The gut microbiome was analyzed using 16 S rRNA amplicon sequencing. Microbial richness was assessed through alpha-diversity measures, including observed features, Simpson, and Shannon indices. Non-metric multidimensional scaling plots were generated using Primer 7 (PRIMER-e, New Zealand) to visualize bacterial composition differences. Random forest analysis was conducted via Microbiome Analyst 2.0 (https://www.microbiomeanalyst.ca/) to identify bacterial genera or taxa that differed between groups.

### Outcome

The primary outcome was to assess hepatic steatosis improvement through anthropometric, biochemical, and MRI changes after 12 weeks of lifestyle intervention, along with assessment of gut microbiota composition. Secondary outcomes analyzed factors affecting MRI fat fraction improvement and the impact of diet and exercise on anthropometric, biochemical parameters, and hepatic steatosis.

### Statistical analyses

Continuous variables were presented as mean ± standard deviation or median with interquartile range and compared using independent t-tests or Mann-Whitney U tests. Categorical variables were analyzed with the Chi-square test. Paired t-tests or Wilcoxon signed-rank tests were used to assess within-group differences. Multiple linear regression with stepwise selection was used to identify independent effects of dietary and physical activity factors on biochemical parameters and hepatic steatosis. All candidate variables were entered into the model, and only those with significant independent associations (*p* < 0.05) were retained in the final model. Gut microbiome analysis included alpha diversity and beta diversity assessment, and area under the receiver-operator curves (AUC) calculations for relative abundance differences. Statistical analysis was performed using SPSS (Version 25; IBM Corp, Armonk, New York).

## Results

### Baseline patient characteristics

A total of 40 patients were recruited between October 2022 and June 2023. After applying predefined exclusion criteria, 31 participants completed the study. All enrolled participants had a BMI Z score ≥ 1 and met at least one cardiometabolic criterion. The median BMI was 28.3 kg/m² (range: 26.8–29.9), corresponding to a median BMI z-score of 1.92 (95% CI, 1.81–2.02), approximately the 96th percentile. The median age was 12 years (range: 10–18), and 26 (83.9%) were male. Median waist circumference was 94.7 cm (range: 90.9–98.5; z-score 1.75). (Table 1) The median AST was 68.9 U/L, ALT was 150.6 U/L, fasting glucose was 102.0 mg/dL, fasting insulin 39.2 mIU/L and triglycerides was 124.5 mg/dL. In liver MRI, the median fat fraction was 27.1% (range: 23.26–30.99). Five patients had mild, ten moderate, and sixteen severe hepatic steatosis.

**Table 1:** Baseline characteristics of study participants

Characteristics	Total (*n* = 31)
Age, y	12 (10–18)
Sex, male/female	26/5
**Anthropometry**	
Height, cm	160.90 (156.40 to 165.41)
Height, Z-score	1.25 (0.37 to 2.13)
Weight, kg	74.61 (67.42 to 81.81)
Weight, Z-score	2.11 (1.90 to 2.33)
BMI, kg/m^2^	28.30 (26.76 to 29.85)
BMI, Z-score	1.92 (1.81 to 2.02)
Systolic blood pressure, mmHg	121.97 (117.57 to 126.37)
Systolic blood pressure, Z-score	1.09 (0.77 to 1.42)
Diastolic blood pressure, mmHg	72.97 (69.55 to 76.38)
Diastolic blood pressure, Z-score	0.75 (0.49 to 1.02)
Waist circumference, cm	94.69 (90.85 to 98.52)
Waist circumference, Z-score	1.75 (1.66 to 1.83)
Physical activity, MET	3731.91 (2902.04 to 4561.78)
Physical activity, pedometer	7805.48 (6890.62 to 8720.34)
**Biochemistry**	
AST, U/L	68.94 (50.31 to 87.56)
ALT, U/L	150.58 (111.41 to 189.75)
ALP, U/L	245.58 (201.67 to 289.49)
GGT, U/L	55.90 (40.55 to 71.26)
Fasting glucose, mg/dL	101.97 (95.78 to 108.16)
Fasting insulin, mIU/L	39.22 (30.07 to 48.37)
Total cholesterol, mg/dL	178.23 (166.54 to 189.91)
HDL-cholesterol, mg/dL	44.45 (41.94 to 46.96)
LDL-cholesterol, mg/dL	126.03 (114.31 to 137.76)
Triglycerides, mg/dL	124.48 (105.93 to 143.04)
HbA1c	5.77 (5.50 to 6.04)
HOMA-IR	9.90 (7.44 to 12.35)
APRI	0.57 (0.36 to 0.78)
Fatty liver index	57.84 (48.19 to 67.48)
Hepatic steatosis index	46.04 (43.87 to 48.22)
MRI liver fat, %	27.13 (23.26 to 30.99)
**Steatosis**	
6.4–17.4%	5 (16.1%)
17.4–22.1%	10 (32.3%)
>22.1%	16 (51.6%)
MRI elastography (kPa)	2.03 (1.89 to 2.16)

Continuous variables were presented as mean ± standard deviation or median (interquartile range) and were compared using an unpaired t-test or Mann-Whitney U test, as appropriate. Categorical variables were compared using the chi-square test. *ALP*, alkaline phosphatase; *ALT*, alanine aminotransaminase; *APRI*, AST to Platelet Ratio Index; *AST*, aspartate aminotransferase; *BMI*, body mass index; *GGT*, γ-glutamyl transferase; *HDL*, high-density lipoprotein; *HOMA-IR*, Homeostatic Model Assessment for Insulin Resistance; *LDL*, low-density lipoprotein; *MET*, metabolic equivalent task

### Changes of anthropometric and biochemical parameters with lifestyle modification

The extent of lifestyle modification over 12 weeks for participants in this study is quantified in Supplementary Table 1. First, based on the daily recommended intake adjusted for height and activity level, 27 participants (87.1%) consumed below the recommended intake, while 4 participants (12.9%) consumed above the recommended intake. Regarding exercise volume, the daily step count showed that 4 participants (12.9%) averaged less than 5000 steps per day, 9 participants (29.0%) averaged between 5000 and 7500 steps, 12 participants (38.7%) averaged between 7500 and 10,000 steps, and 6 participants (19.4%) averaged more than 10,000 steps per day. In terms of MET, no participants were below 600 MET, 16 participants (51.6%) were between 600 and 3000 MET, and 15 participants (48.4%) were above 3000 MET.

**Fig. 1:**
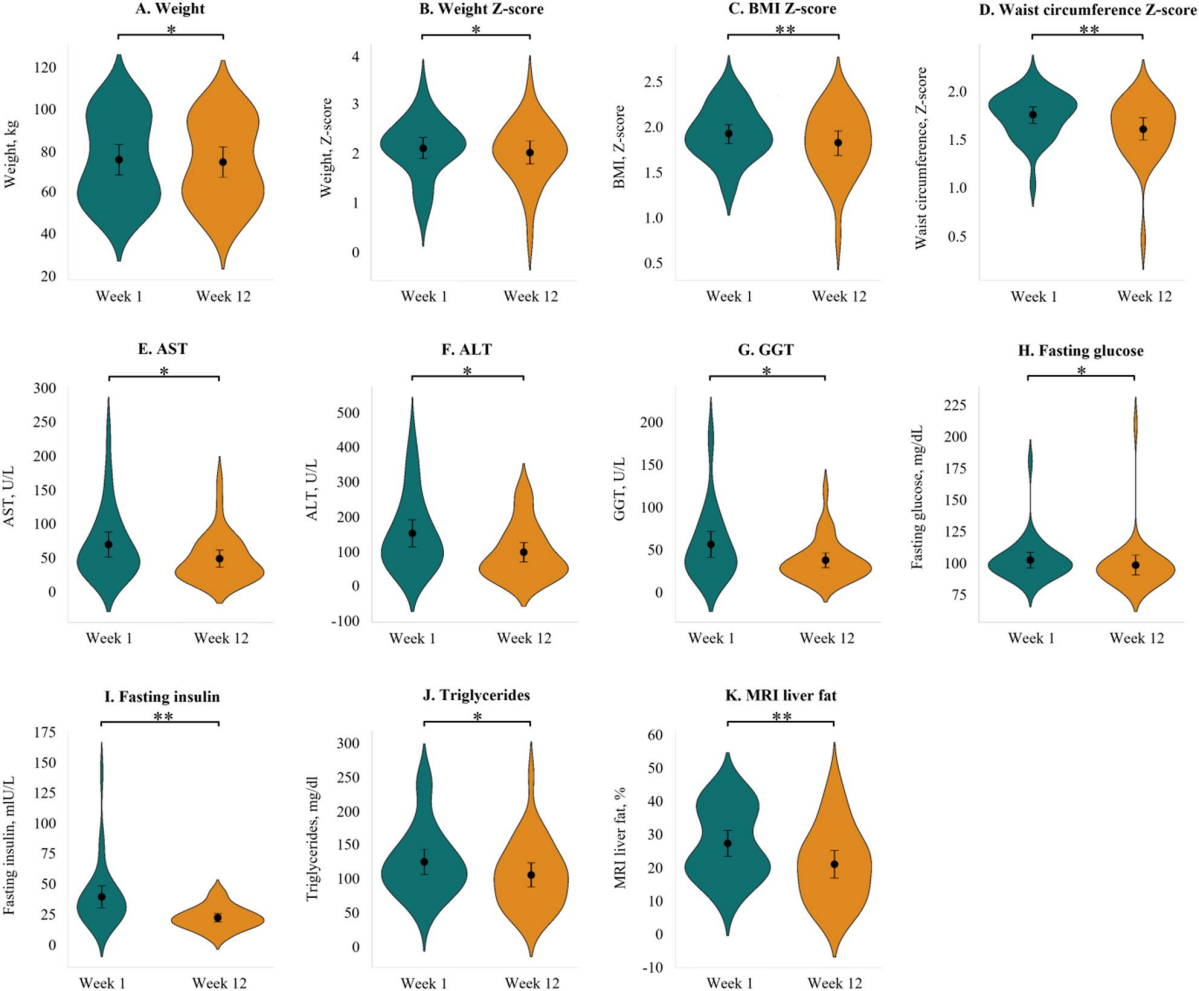
Changes of anthropometric and biochemical parameters at baseline and the end of the study. **a**. body weight Z score; **b**. BMI Z score; **c**. waist circumference Z score; d. AST; **e**. ALT; **f**. GGT; **g**. fasting glucose; **h**. fasting insulin; **i.** triglycerides; **j**. MRI liver fat. ALP, alkaline phosphatase; ALT, alanine aminotransaminase; AST, aspartate aminotransferase; BMI, body mass index; GGT, γ-glutamyl transferase. *P < 0.05, **P < 0.01

After the intervention, significant improvements were observed in anthropometric, biochemical, and liver MRI results. (Fig. 1 and Supplementary Table 2) Median body weight Z-score, BMI Z-score, waist circumference Z-score, ALT, GGT, fasting glucose, fasting insulin, and triglycerides all showed statistically significant reductions. Additionally, liver MRI revealed a significant decrease in hepatic fat fraction in 23 patients (74.2%), from 27.1% to 20.8%. Steatosis grade also improved in 24 participants (77.4%), and three children (9.7%) achieved normalization of liver fat (< 6.4%), indicating resolution of hepatic steatosis.

### Clinical correlates of hepatic fat reduction

Subgroup analysis revealed significant differences in baseline body weight Z-score, BMI Z-score, diastolic blood pressure Z-score, and waist circumference Z-score between participants with and without ≥ 30% relative reduction in MRI-PDFF (Supplementary Table 3) As summarized in Supplementary Table 4, participants achieving ≥ 30% relative reduction in MRI-PDFF also exhibited significantly higher levels of physical activity (steps and METs) and a greater dietary protein proportion. Weight change correlated with liver fat reduction (*r* = 0.579, *p* = 0.001), and participants who achieved ≥ 7% weight loss showed significantly greater reductions in hepatic fat (*p* < 0.05). (Supplementary Fig. 1)

### Impact of amount of exercise and dietary modification on treatment response

Protein intake demonstrated negative standardized beta coefficients with respect to AST and ALT levels (β = −0.367 and − 0.355, respectively) (Table 2), suggesting a beneficial association. Carbohydrate intake was positively associated with total cholesterol and LDL-cholesterol (β = 0.413 and 0.408). Increased daily step count and higher protein intake significantly contributed to hepatic fat reduction, explaining 45.3% of the variance (β = −0.622 and − 0.457, respectively), with physical activity having the larger effect. An increase of 1,000 daily steps was associated with an approximate 2% reduction in MRI-measured hepatic fat fraction (B = − 0.002, *p* = 0.0019), while each 1% higher dietary protein proportion was associated with a 1.4% lower MRI-PDFF (B = − 1.44, *p* = 0.0025).

**Table 2:** The association of lifestyle factors with biochemical parameters and hepatic steatosis at the end of the study in patients with MASLD

Dependent variable	*R* ^2^	Adjusted *R*^2^	Durbin-Watson	Independent variable	B	β	t	*P*-value	VIF
Change in AST, U/L	0.135	0.105	1.639	Constant	91.035				
				**Protein ratio**	**−6.296**	**−0.367**	**−2.127**	**0.042**	1
Change in ALT, U/L	0.126	0.096	1.57	Constant	204.149				
				**Protein ratio**	**−14.568**	**−0.355**	**−2.047**	**0.0498**	1
Change in total cholesterol, mg/dL	0.17	0.142	1.745	Constant	−99.441				
				**Carbohydrate ratio**	**1.822**	**0.413**	**2.44**	**0.021**	1
Change in LDL- cholesterol, mg/dL	0.167	0.138	1.683	Constant	−101.75				
				**Carbohydrate ratio**	**1.862**	**0.408**	**2.409**	**0.023**	1
Change in MRI fat fraction, %	0.49	0.453	1.696	Constant	32.642				
				**Pedometer**	**−0.002**	**−0.622**	**−4.528**	**0.0019**	1.036
				**Protein ratio**	**−1.438**	**−0.457**	**−3.326**	**0.0025**	1.036

Analysis was done by multiple linear regression. Bold values indicate statistically significant associations (*p* < 0.05). *ALT*, alanine aminotransaminase; *AST*, aspartate aminotransferase; *LDL*, low-density lipoprotein; *MET*, metabolic equivalent task; *VIF*, variance inflation factor

### Fecal microbiota analysis

**Fig. 2:**
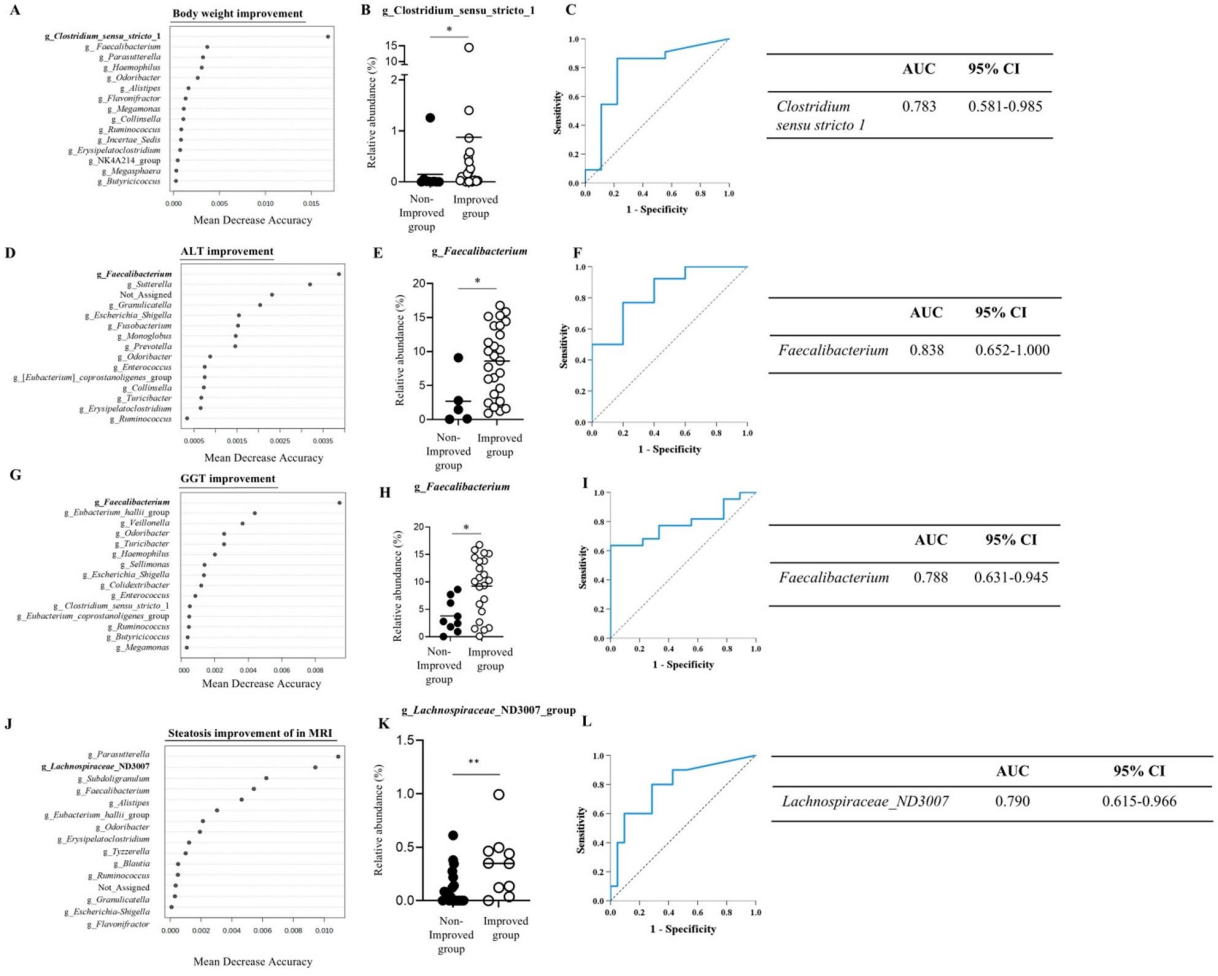
Baseline gut microbiome discriminated participants with clinical improvements. **a**. Random Forest analysis identifying genera associated with body weight reduction; **b**. Relative abundance of significant genera by body weight change; **c.** ROC curve for *Clostridium sensu stricto 1*; **d**. Random Forest analysis identifying genera associated with ALT improvement; **e**. Relative abundance of significant genera by ALT change; **f.** ROC curve for *Faecalibacterium*; **g**. Random Forest analysis identifying genera associated with GGT improvement; **h**. Relative abundance of significant genera by GGT change; **i.** ROC curve for *Faecalibacterium*;**j.** Random Forest analysis identifying genera associated with steatosis improvement in MRI; **k**. Relative abundance of significant genera by steatosis improvement in MRI; **l.** ROC curve for *Lachnospiraceae_ND3007_group*. *ALT*,* alanine aminotransaminase*; *GGT*,* γ-glutamyl transferase;*Mann-Whitney U test was performed. *P < 0.05, **P < 0.01

No significant changes in alpha or beta diversity were observed (Supplementary Figs. 2–3), and microbial composition showed no meaningful differences according to dietary macronutrient categories. (Supplementary Table 5) However, baseline gut microbiome composition differed between participants with and without clinical improvements. Significant weight loss was associated with higher abundance of *Clostridium sensu stricto 1*, which remained significant after FDR correction. (*p* = 0.015, *q* = 0.046) (Fig. 2A, B). *Faecalibacterium* was enriched at baseline in participants with improved ALT and GGT levels, and retained significance following FDR adjustment (ALT: *p* = 0.015, *q* = 0.046; GGT: *p* = 0.014, *q* = 0.042) (Fig. 2D, E, G, H). For those with ≥ 30% reduction in hepatic fat fraction on MRI, *Lachnospiraceae_ND3007_group* showed a significant association after FDR correction (*q* = 0.018) (Fig. 2J, K). Baseline microbiome composition discriminated participants with clinical improvements, with all comparisons achieving AUC ≥ 0.75 (Fig. 2C, F, I, L). In contrast, microbial differences observed after the intervention in participants with clinical improvements did not remain significant after FDR correction, and are presented in Supplementary Fig. 4. Baseline microbiome associations and corresponding FDR-adjusted statistics are detailed in Supplementary Table 6.

## Discussion

HEALKIDS is the first study to assess the effects of lifestyle modification on anthropometrics, biochemistry, liver fat fraction, and gut microbiota in pediatric MASLD. After a 12–week exercise and dietary adjustments, significant improvements were observed in body weight, BMI, waist circumference, liver enzymes, fasting glucose, insulin, triglycerides, and MRI–measured liver fat. Protein intake positively influenced AST and ALT improvement, while both protein intake and daily steps contributed to MRI liver fat reduction. In microbiome analysis, baseline gut microbial composition differed between clinical responder groups. *Clostridium sensu stricto 1* was enriched at baseline in participants with significant weight loss, *Faecalibacterium* in those with ALT or GGT improvement, and *Lachnospiraceae_ND3007_group* in those with MRI-PDFF reduction.

MASLD is linked to higher all-cause, cardiac, and cancer mortality, with NASH-related hepatocellular carcinoma becoming a growing public health concern [15, 16]. Both aerobic and resistance training improve liver fat, and a 5–7% weight loss can reverse fatty liver [17, 18]. In this study, lifestyle modification led to significant improvements in anthropometric and biochemical parameters, HOMA-IR, APRI, FLI, and MRI–measured hepatic fat, supporting the effectiveness of lifestyle intervention. Similar studies in pediatric MASLD showed improvements in weight, ALT, and HOMA-IR after 6 months of intensive lifestyle management [19]. In adult, a 12-week aerobic exercise program improved body mass and waist circumference [20], while 8–week and 20–week interventions significantly improved liver enzymes, FLI, APRI, and MRI liver fat in MASLD and NASH patients [7, 10]. 

In pediatric MASLD, a controlled feeding randomized trial demonstrated that intensive sugar restriction leads to meaningful reductions in liver fat, as assessed by MRI-PDFF [21]. In contrast, our study assessed diet using three-day food diaries rather than controlled feeding, which may have limited the accuracy of sugar quantification and contributed to the lack of differences in sugar intake between responders and non-responders.

Several exercise-based pediatric studies have shown improvements in BMI, triglycerides, and liver enzymes following structured physical activity programs, although these trials did not directly measure hepatic fat [22, 23]. In our study, higher physical activity—predominantly aerobic exercise—was associated with greater reductions in MRI-measured hepatic fat. This aligns with a recent network meta-analysis identifying aerobic exercise as the most effective modality for improving metabolic outcomes in NAFLD, as well as RCT evidence showing direct hepatic fat reduction following structured aerobic training [24, 25]. However, most supporting evidence is derived from adult MASLD populations, and extrapolation to pediatric patients should be interpreted with caution.

Weight loss and reduced waist circumference are strongly associated decreased liver fat [9, 26]. In this study, weight and waist circumference Z-score influenced MRI fat fraction improvement. A 7–10% weight loss can reverse MASLD in most patients [27, 28], and those achieving > 7% weight loss showed significant MRI liver fat improvement, aligning with previous findings.

MASLD patients are generally advised to follow a Mediterranean-style diet that limits simple carbohydrates and saturated fat while ensuring adequate protein intake [18, 29]. In our study, higher protein intake was associated with improvements in AST and ALT, whereas lower carbohydrate intake was associated with favorable changes in total and LDL-cholesterol. A previous pediatric study similarly reported lower protein intake in children with MASLD [30], and our findings extend this by showing that a higher protein proportion was also associated with MRI-measured reductions in hepatic fat. Moreover, greater daily step counts were associated with larger improvements in MRI-PDFF, underscoring the combined importance of diet quality and habitual physical activity in pediatric MASLD [31–33]. 

Several studies have reported reduced alpha and beta diversity in MASLD patients [34, 35], with partial recovery following lifestyle interventions [7]. However, our study did not observe significant changes in microbial diversity overall, likely reflecting the strong inter–individual variability of gut microbiome, which may exceed biochemical variation and remain stable despite dietary changes [36]. 

Baseline microbiome profiles were associated with clinical outcome. Several studies have reported that *Clostridium sensu stricto 1* abundance decreases with the progression of MASLD, particularly in relation to hepatic steatosis and fibrosis [37, 38]. In our study, participants who achieved clinically meaningful weight loss showed significantly higher baseline abundance of *Clostridium sensu stricto 1*, suggesting that the relative abundance of this taxon may be associated with metabolic improvement in MASLD. *Faecalibacterium* was enriched in participants with improved ALT and GGT, aligning with previous studies reporting its depletion in MASLD and restoration alongside improvements in liver enzymes [39, 40] This observation is also consistent with evidence that the Mediterranean diet—known to benefit MASLD—increases *Faecalibacterium* abundance [18, 41].

Similarly, recent studies have consistently reported that the *Lachnospiraceae ND3007* group is significantly reduced in patients with MASLD, with lower levels linked to hepatic fat accumulation and elevated liver enzymes such as ALT and AST [42–44]. This taxon is positively associated with fiber-rich, plant-based diets and is a known producer of short-chain fatty acids (SCFAs) through dietary fiber fermentation [45]. In our study, participants who achieved a ≥ 30% reduction in hepatic fat content on MRI had significantly higher baseline abundance of the *Lachnospiraceae ND3007* group, supporting its potential relevance in MASLD improvement. Although no studies have directly examined whether baseline gut microbiota predicts treatment response, prior work has demonstrated its prognostic value for disease severity and future liver disease risk [46, 47]. Building on this background, our findings suggest that specific baseline taxa may help identify individuals more likely to benefit from lifestyle interventions in MASLD.

This study has several limitations. First, it was a single-arm interventional study without a control group, which limits the interpretation of the results and causal inference. Although ethical concerns make placebo-controlled trials difficult to perform in pediatric populations, this study was designed as a pilot feasibility study to explore preliminary metabolic and microbiome changes associated with lifestyle modification. Second, being a single–center study with a small sample size, statistical power was limited in assessing lifestyle effects and gut microbiota changes, making it difficult to confirm associations between diet, physical activity, and bacterial genera. Third, classification of participants with improved ALT or GGT levels was exploratory, defined as those showing a ≥ 10% relative reduction from baseline to week 12. This threshold was chosen empirically to account for known biological variability in liver enzyme measurements. In addition, the MRI-PDFF response threshold (≥ 30% relative reduction) was adopted from adult studies with biopsy-proven NASH; pediatric-specific validation data remain limited, and this cutoff should therefore be interpreted with caution in children. Fourth, MASLD diagnosis relied on MRI instead of liver biopsy, preventing the evaluation of histological features. However, MRI is recognized for assessing MASLD severity [2], and liver fat fraction was accurately measure in this study. Fifth, food diaries were collected only after the initial nutrition counseling, preventing assessment of baseline dietary habits. Although total sugar intake was recorded during the intervention, the diary format did not allow accurate quantification of added sugars, limiting our ability to evaluate sugar-related effects. Nevertheless, dietary adherence and macronutrient distribution during the 12-week intervention were assessed. Lastly, the study had a sex imbalance (26 males, 5 females). While sex-specific microbiome difference in MASLD have been reported [48], this pre–post study design minimizes impact on stool analysis.

Despite limitations, this study has several strengths. It is the first to comprehensively analyze the effects of exercise and diet, as recommended by clinical guidelines [49], on anthropometrics, biochemistry, hepatic fat, and gut microbiota in pediatric MASLD. Although the 12–week period was short, significant improvements were observed in body weight, waist circumference, liver enzyme, and hepatic fat fraction, highlighting the importance of macronutrient balance and physical activity in fatty liver improvement. Baseline gut microbiota composition differed in participants with improvements in ALT, GGT, and MRI-PDFF, suggesting that pre-intervention microbial profiles may be associated with clinical response. Specific microbial taxa at baseline also showed potential as predictors of liver health improvement. Additionally, systematic evaluation and wearable devices likely contributed to adherence and positive behavior changes [50, 51]. 

In conclusion, a 12-week lifestyle intervention improved anthropometric measures, biochemical parameters, and hepatic steatosis in pediatric MASLD, and was associated with distinct baseline gut microbial profiles in clinical responders. Improvements in hepatic steatosis were associated with reductions in weight, BMI, and waist circumference, as well as higher physical activity and protein intake. Notably, patients with > 7% weight loss showed significantly greater hepatic fat reduction. These findings support the effectiveness and safety of individualized lifestyle programs for managing pediatric MASLD. Larger randomized controlled trials are warranted to validate these results.

## Supplementary Information

Supplementary Material 1.

Supplementary Material 2.

## Data Availability

The datasets generated and analyzed during this study, including the redacted study protocol, redacted statistical analysis plan, and individual participant-level data supporting the reported results, will be made available upon reasonable request. Data will be shared with researchers who provide a methodologically sound proposal and will be de-identified in compliance with applicable privacy laws, data protection regulations, and requirements for consent and anonymization.All raw 16 S rRNA amplicon sequences derived from this experiment were submitted to the Short Read Archive of NCBI and can be accessed under the BioProject accession number PRJNA1103392 (https://www.ncbi.nlm.nih.gov/bioproject/1103392).

## Abbreviations

APRI: AST to Platelet Ratio Index
HOMA-IR: Homeostatic Model Assessment for Insulin Resistance
MASLD: Metabolic dysfunction-associated steatotic liver disease
NAFLD: Non-alcoholic fatty liver disease
