# Supplementary material for: Clinical and microbial correlates of response to lifestyle intervention in pediatric metabolic dysfunction-associated steatotic liver disease
Source: Gut Pathog. 2026 Jan 18;18:6. doi: 10.1186/s13099-026-00798-5 (PMC12831411; doi:10.1186/s13099-026-00798-5)
Supplement: Supplementary file 1 — Supplementary Material 1. [file 13099_2026_798_MOESM1_ESM.docx]

**Supplementary Appendix**

**Clinical and Microbial correlates of response to lifestyle intervention in pediatric metabolic dysfunction-associated steatotic liver disease**

Jong Woo Hahn MD, PhD^1,2^, Jin Gyu Lim MD^1^, Kyung Jae Lee MD, PhD^1^, Jin Soo Moon MD, PhD^1^, Tae Hyeong Kim MD^3^, Yejun Son^4^, Dong Keon Yon MD, PhD^4,5^, Yun Jung Lee^6^, Yuri Seo^6^, Jihyun Park^7^, Seunghyun Lee MD, PhD^8*^, Donghyun Kim, PhD^7,9*^ and Jae Sung Ko MD, PhD^1*^

^1^Department of Pediatrics, Seoul National University College of Medicine, Seoul, South Korea

^2^Department of Pediatrics, Seoul National University Bundang Hospital, Seongnam, Gyeonggi-do, South Korea

^3^Department of Pediatrics, Kyung Hee University Hospital at Gangdong, Seoul, Korea.

^4^Center for Digital Health, Medical Science Research Institute, Kyung Hee University College of Medicine, Seoul, South Korea

^5^Department of Pediatrics, Kyung Hee University Medical Center, Kyung Hee University College of Medicine, Seoul, South Korea

^6^Department of Food Service and Nutrition Care, Seoul National University Hospital, Seoul, South Korea.

^7^Department of Biomedical Sciences, Seoul National University College of Medicine, Seoul, South Korea

^8^Department of Radiology, Seoul National University College of Medicine, Seoul, South Korea

^9^Department of Microbiology and Immunology, Seoul National University College of Medicine, Seoul, South Korea

**Correspondence**

Jae Sung Ko, MD, PhD

Professor

Department of Pediatrics

Seoul National University College of Medicine

Seoul National University Children's Hospital

101 Daehak-ro, Jongno-Gu, Seoul, 03080, Korea

Tel; 82-2-2072-2197, Fax; 82-2-743-3455

E-mail : [kojs@snu.ac.kr](mailto:kojs@snu.ac.kr)

Seunghyun Lee MD, PhD

Department of Radiology

Seoul National University College of Medicine

Seoul National University Children's Hospital

101 Daehak-ro, Jongno-Gu, Seoul, 03080, Korea

Tel; 82-2-2072-3608 , Fax; 82-2-747-5781

E-mail : [sh22@snu.ac.kr](mailto:sh22@snu.ac.kr)

Donghyun Kim, PhD

Department of Microbiology and Immunology

Seoul National University College of Medicine

101 Daehak-ro, Jongno-Gu, Seoul, 03080, Korea

Tel; 82-2-740-8304, Fax; 82-2-743-0881

E-mail : [biologokim@snu.ac.kr](mailto:kojs@snu.ac.kr)

**Contents of supplementary appendix**

| Supplementary Table 1 | Extent of dietary control and exercise over 12 weeks in this study. | Page 6 |
| --- | --- | --- |
| Supplementary Table 2 | Changes of anthropometric and biochemical parameters at baseline and the end of the study | Page 7 |
| Supplementary Table 3 | Factors associated with improvement in liver fat content (≥30% relative reduction in MRI-PDFF after 12 weeks of lifestyle modification). | Page 9 |
| Supplementary Table 4 | Physical activity and dietary adherence according to MRI-PDFF response (≥30% relative reduction vs <30%). | Page 11 |
| Supplementary Table 5 | Exploratory analysis of gut microbial composition according to dietary intake categories | Page 12 |
| Supplementary Table 6 | FDR-adjusted p-values for gut microbial genera with the highest discriminatory contribution to baseline microbiota differences between participants with and without clinical improvement. | Page 15 |
| Supplementary Figure 1. | Correlation between relative changes in body weight and MRI-measured hepatic fat fraction | Page 16 |
| Supplementary Figure 2. | Beta diversity (NMDS plot) of non-responders and responders about each clinical index before and after exercise-intervention. | Page 17 |
| Supplementary Figure 3. | Alpha diversity (observed Features, Simpson index, and Shannon index) of non-responders and responders about each clinical index before and after exercise-intervention. | Page 18 |
| Supplementary Figure 4. | Gut microbial genera exhibiting a significant difference in the abundance before and after exercise-intervention | Page 19 |

| Supplementary Table 1. Extent of dietary control and exercise over 12 weeks in this study.   \| Variables \| Classification \| n (%) \| \| --- \| --- \| --- \| \| Total average daily intake, kcal \| Less than the recommended total average daily intake \| 27 (87.1%) \| \|  \| More than the recommended total average daily intake \| 4 (12.9%) \| \| Carbohydrate ratio, % \| Below 55% of carbohydrate in the total average daily intake \| 23 (74.2%) \| \|  \| over 55% of carbohydrate in the total average daily intake \| 8 (25.8%) \| \| Protein ratio, % \| Below 20% of protein in the total average daily intake \| 25 (80.6%) \| \|  \| Over 20% of protein in the total average daily intake \| 6 (19.4%) \| \| Fat ratio, % \| Below 30% of fat in the total average daily intake \| 12 (38.7%) \| \|  \| Over 30% of fat in the total average daily intake \| 19 (61.3%) \| \| Pedometer, average daily steps \| <5000 \| 4 (12.9%) \| \|  \| 5000-7500 \| 9 (29.0%) \| \|  \| 7500-10000 \| 12 (38.7%) \| \|  \| >10000 \| 6 (19.4%) \| \| Metabolic Equivalents of Task score in week \| 600-3000 \| 16 (51.6%) \| \|  \| >3000 \| 15 (48.4%) \| |
| --- | --- | --- | --- | --- | --- | --- | --- | --- | --- | --- | --- | --- | --- | --- | --- | --- | --- | --- | --- | --- | --- | --- | --- | --- | --- | --- | --- | --- | --- | --- | --- | --- | --- | --- | --- | --- | --- | --- | --- | --- | --- | --- | --- | --- | --- |

| Supplementary Table 2. Changes of anthropometric and biochemical parameters at baseline and the end of the study. | | | | |
| --- | --- | --- | --- | --- |
| Variables | Baseline (Week 0) | End of Study (Week 12) | Difference between Week 0 and Week 12 | |
|  |  |  | Mean difference (95% CI) | *P* value |
| Weight, kg | 75.29 (67.97 to 82.60) | 73.50 (66.37 to 80.62) | **-1.79 (-2.96 to -0.62)** | **0.004** |
| Weight, Z-score | 2.11 (1.90 to 2.33) | 2.02 (1.79 to 2.26) | **-0.09 (-0.15 to -0.03)** | **0.007** |
| BMI, kg/m^2^ | 28.30 (26.76 to 29.85) | 27.57 (25.93 to 29.22) | **-0.89 (-1.33 to -0.45)** | **<.001** |
| BMI, Z-score | 1.92 (1.81 to 2.02) | 1.82 (1.68 to 1.95) | **-0.11 (-0.16 to -0.05)** | **<.001** |
| Systolic blood pressure, mmHg | 121.97 (117.57 to 126.37) | 118.67 (115.38 to 121.96) | -3.27 (-7.01 to 0.48) | 0.085 |
| Systolic blood pressure, Z-score | 1.09 (0.77 to 1.42) | 0.74 (0.49 to 0.99) | -0.34 (-0.70 to 0.02) | 0.064 |
| Diastolic blood pressue, mmHg | 72.97 (69.55 to 76.38) | 74.07 (71.45 to 76.68) | 1.37 (-1.67 to 4.40) | 0.365 |
| Diastolic blood pressure, Z-score | 0.75 (0.49 to 1.02) | 0.81 (0.62 to 1.00) | 0.08 (-0.18 to 0.34) | 0.541 |
| Waist circumference, cm | 94.69 (90.85 to 98.52) | 91.34 (87.38 to 95.29) | **-3.75 (-4.97 to -2.52)** | **<.001** |
| Waist circumference, Z-score | 1.75 (1.66 to 1.83) | 1.60 (1.49 to 1.72) | **-0.15 (-0.21 to -0.09)** | **<.001** |
| AST, U/L | 68.94 (50.31 to 87.56) | 48.16 (35.54 to 60.78) | **-20.77 (-34.52 to -7.03)** | **0.004** |
| ALT, U/L | 150.58 (111.41 to 189.75) | 96.00 (68.00 to 124.00) | **-54.58 (-87.46 to -21.70)** | **0.002** |
| GGT, U/L | 55.90 (40.55 to 71.26) | 37.35 (28.62 to 46.09) | **-18.55 (-29.83 to -7.27)** | **0.002** |
| Fasting glucose, mg/dL | 101.97 (95.78 to 108.16) | 98.03 (90.14 to 105.93) | **-3.94 (-7.61 to -0.26)** | **0.037** |
| Fasting insulin, mIU/L | 39.22 (30.07 to 48.37) | 22.01 (18.52 to 25.50) | **-17.21 (-26.10 to -8.31)** | **<.001** |
| Total cholesterol, mg/dL | 178.23 (166.54 to 189.91) | 171.94 (159.99 to 183.89) | -6.29 (-16.09 to 3.51) | 0.200 |
| HDL cholesterol, mg/dL | 44.45 (41.94 to 46.96) | 45.29 (42.20 to 48.38) | 0.84 (-0.73 to 2.41) | 0.283 |
| LDL cholesterol, mg/dL | 126.03 (114.31 to 137.76) | 119.48 (106.99 to 131.97) | -6.55 (-16.67 to 3.58) | 0.197 |
| Triglycerides, mg/dL | 124.48 (105.93 to 143.04) | 105.13 (87.34 to 122.92) | **-19.35 (-35.36 to -3.35)** | **0.019** |
| HbA1c | 5.77 (5.50 to 6.04) | 5.68 (5.36 to 5.99) | **-0.09 (-0.18 to -0.01)** | **0.040** |
| HOMA-IR | 9.90 (7.44 to 12.35) | 5.31 (4.43 to 6.20) | **-4.58 (-7.06 to -2.10)** | **<.001** |
| APRI | 0.57 (0.36 to 0.78) | 0.40 (0.27 to 0.53) | **-0.17 (-0.30 to -0.05)** | **0.008** |
| Fatty liver index | 57.84 (48.19 to 67.48) | 47.43 (36.50 to 58.37) | **-11.53 (-15.78 to -7.29)** | **<.001** |
| Hepatic steatosis index | 46.04 (43.87 to 48.22) | 42.91 (39.94 to 45.89) | **-3.28 (-4.66 to -1.90)** | **<.001** |
| MRI liver fat, % | 27.13 (23.26 to 30.99) | 20.84 (16.71 to 24.96) | **-6.29 (-8.81 to -3.77)** | **<.001** |
| MRI elastography, kPa | 2.03 (1.89 to 2.16) | 2.04 (1.89 to 2.19) | 0.01 (-0.08 to 0.10) | 0.816 |

Continuous variables were presented as mean ± standard deviation or median (interquartile range) and were compared using an unpaired t-test or Mann-Whitney U test, as appropriate. Categorical variables were compared using the chi-square test. Values in bold designate significance. *ALP*, alkaline phosphatase; *ALT*, alanine aminotransaminase; *APRI*, AST to Platelet Ratio Index; *AST*, aspartate aminotransferase; *BMI*, body mass index; *GGT*, γ-glutamyl transferase; *HOMA-IR*, Homeostatic Model Assessment for Insulin Resistance; *MET*, metabolic equivalent task.

Supplementary Table 3. Factors associated with improvement in liver fat content (≥30% relative reduction in MRI-PDFF after 12 weeks of lifestyle modification).

| Variables | No improvement in MRI liver fat (<30% reduction, n=21) | Improvement in MRI liver fat (≥30% reduction, n=10) | *P* value |
| --- | --- | --- | --- |
|  |  |  |  |
| Change in body weight, kg | **-0.80 (-2.46 to 0.63)** | **-3.78 (-5.19 to -2.36)** | **0.011** |
| Change in body weight Z score | **-0.23 (-0.09 to 0.04)** | **-0.22 (-0.31 to -0.12)** | **0.001** |
| Change in BMI, kg/m^2^ | **-0.47 (-1.04 to 0.06)** | **-1.74 (-2.31 to -1.17)** | **0.003** |
| Change in BMI Z score | **-0.05 (-0.11 to 0.00)** | **-0.22 (-0.32 to -0.11)** | **0.002** |
| Change in systolic blood pressure, mmHg | -3.15 (-8.90 to 2.17) | -3.50 (-8.93 to 1.93) | 0.930 |
| Change in systolic blood pressure Z score | -0.34 (-0.85 to 0.18) | -0.34 (-0.86 to 0.17) | 0.982 |
| Change in diastolic blood pressure, mmHg | 1.70 (-3.11 to 5.53) | 0.70 (-3.90 to 5.30) | 0.757 |
| Change in diastolic blood pressure Z score | 0.10 (-0.28 to 0.47) | 0.05 (-0.35 to 0.44) | 0.861 |
| Change in waist circumference, cm | -3.21 (-4.69 to -1.46) | -4.82 (-7.02 to -2.62) | 0.210 |
| Change in waist circumference Z score | -0.10 (-0.17 to -0.04) | -0.23 (-0.36 to -0.10) | 0.036 |
| Change in AST, U/L | **-10.19 (-19.99 to 0.62)** | **-43.00 (-81.58 to -4.42)** | **0.020** |
| Change in ALT, U/L | **-24.00 (-43.49 to -4.51)** | **-113.40 (-207.57 to -19.22)** | **0.009** |
| Change in GGT, U/L | -13.38 (-24.13 to -3.13) | -29.40 (-61.31 to 2.51) | 0.179 |
| Change in fasting glucose, mg/dL | -4.52 (-10.65 to 1.28) | -2.70 (-6.22 to 0.82) | 0.644 |
| Change in fasting insulin, mIU/L | -19.05 (-33.81 to -4.74) | -13.33 (-20.72 to -5.94) | 0.548 |
| Change in total cholesterol, mg/dL | -1.95 (-17.77 to 8.40) | -15.40 (-32.59 to 1.79) | 0.195 |
| Change in HDL-cholesterol, mg/dL | 1.14 (-0.85 to 2.43) | 0.20 (-3.98 to 4.38) | 0.575 |
| Change in LDL-cholesterol, mg/dL | 2.10 (-9.65 to 18.92) | 15.90 (0.12 to 31.68) | 0.198 |
| Change in Triglyceride, mg/dL | -22.90 (-48.19 to 1.66) | -11.90 (-33.21 to 9.41) | 0.521 |
| Change in HbA1c | -0.08 (-0.22 to 0.05) | -0.11 (-0.21 to -0.01) | 0.753 |
| Change in HOMA-IR | -5.20 (-9.35 to -1.23) | -3.29 (-5.19 to -1.40) | 0.473 |
| Change in APRI | -0.01 (-0.25 to 0.05) | -0.32 (-0.61 to -0.04) | 0.086 |
| Change in FLI | **-7.95 (-13.35 to -2.02)** | **-18.70 (-23.85 to -13.55)** | **0.012** |
| Change in HSI | **-1.69 (-3.09 to -0.06)** | **-6.46 (-8.32 to -4.60)** | **<0.001** |

Continuous variables were presented as mean ± standard deviation or median (interquartile range) and were compared using an unpaired t-test or Mann-Whitney U test, as appropriate. Categorical variables were compared using the chi-square test. Values in bold designate significance. *ALP*, alkaline phosphatase; *ALT*, alanine aminotransaminase; *APRI*, AST to Platelet Ratio Index; *AST*, aspartate aminotransferase; *BMI*, body mass index; *FLI*, fatty liver index; *GGT*, γ-glutamyl transferase; *HDL*, high-density lipoprotein; *HOMA-IR*, Homeostatic Model Assessment for Insulin Resistance; *HSI*, hepatic steatosis index; *LDL*, low-density lipoprotein; *MET*, metabolic equivalent task.

Supplementary Table 4. Physical activity and dietary adherence according to MRI-PDFF response (≥30% relative reduction vs <30%).

| Variables | No improvement in MRI liver fat (<30% reduction, n=21) | Improvement in MRI liver fat (≥30% reduction, n=10) | *P* value |
| --- | --- | --- | --- |
|  |  |  |  |
| Metabolic Equivalents of Task score in week | **3164.98 (2244.28 to 4085.68)** | **4922.46 (3222.69 to 6622.23)** | **0.041** |
| Pedometer, average daily steps | **7023.95 (5991.06 to 8056.54)** | **9446.71 (7867.11 to 11026.30)** | **0.009** |
| Total average daily intake, kcal | 1994.59 (1800.65 to 2188.53) | 1931.86 (1606.05 to 2257.67) | 0.710 |
| Carbohydrate ratio, % | 52.24 (49.40 to 55.09) | 48.76 (45.09 to 52.43) | 0.136 |
| Protein ratio, % | **17.15 (16.16 to 18.15)** | **19.03 (17.87 to 20.19)** | **0.022** |
| Fat ratio, % | 30.60 (28.26 to 32.95) | 32.21 (29.18 to 35.23) | 0.399 |
| Total sugar intake, g | 34.35 (27.76 to 40.93) | 35.20 (26.33 to 44.07) | 0.874 |

Continuous variables were presented as mean ± standard deviation or median (interquartile range) and were compared using an unpaired t-test or Mann-Whitney U test, as appropriate. Categorical variables were compared using the chi-square test. Values in bold designate significance.

Supplementary Table 5. Exploratory analysis of gut microbial composition according to dietary intake categories.

Supplementary Table 5A. Pre–post changes in selected genera according to carbohydrate intake (≤55% vs >55% of total energy)

| **Genus** | **Intake group analyzed** | **Pre–post change within group** | **Statistical significance** | **Interpretation** |
| --- | --- | --- | --- | --- |
| *Eggerthella* | Low-carbohydrate (≤55%) | No meaningful change | Not significant | No evidence of carbohydrate-related impact |
| *Escherichia–Shigella* | Low-carbohydrate (≤55%) | Decrease observed | Statistically significant, but driven by baseline differences | Higher baseline abundance limits interpretability |
| *Eubacterium_hallii group* | High-carbohydrate (>55%) | No meaningful change | Not significant | Highly abundant genus; no intervention effect |

Carbohydrate intake was classified as ≤55% vs >55% of total daily energy intake. Candidate genera were selected from random forest variable importance plots. Paired pre–post comparisons were conducted within each intake group using Wilcoxon signed-rank tests. A statistically significant decrease in *Escherichia–Shigella* in the low-carbohydrate group was observed; however, this appeared attributable to higher baseline abundance in this group, limiting interpretability. No other genera showed significant pre–post changes.

Supplementary Table 5B. Pre–post changes in selected genera according to protein intake (≤20% vs >20% of total energy)

| **Genus** | **Intake group analyzed** | **Pre–post change within group** | **Statistical significance** | **Interpretation** |
| --- | --- | --- | --- | --- |
| *Lactobacillus* | Low-protein (≤20%) | Baseline difference observed; post-intervention difference disappeared | Not significant | Implies baseline imbalance rather than intervention effect |
| *Escherichia–Shigella* | Low-protein (≤20%) | Slight decrease trend | Not significant | No consistent protein-related effect |
| *Subdoligranulum* | High-protein (>20%) | No meaningful change | Not significant | — |
| *Streptococcus* | High-protein (>20%) | No meaningful change | Not significant | — |
| *Bacteroides* | High-protein (>20%) | No meaningful change | Not significant | — |
| *Flavonifractor* | High-protein (>20%) | No meaningful change | Not significant | — |

Protein intake was classified as ≤20% versus >20% of total daily energy intake. Candidate genera identified from random forest variable importance were evaluated using Wilcoxon signed-rank tests within each intake group, and no genera showed statistically significant pre–post changes.

Supplementary Table 5C. Pre–post changes in selected genera according to fat intake (≤30% vs >30% of total energy)

| **Genus** | **Intake group analyzed** | **Pre–post change within group** | **Statistical significance** | **Interpretation** |
| --- | --- | --- | --- | --- |
| *Paraprevotella* | Low-fat (≤30%) | No meaningful change | Not significant | No evidence of fat-related microbiome effect |
| *Alistipes* | Low-fat (≤30%) | No meaningful change | Not significant | — |
| — | High-fat (>30%) | No candidate genera identified; no paired comparison performed | — | No genera showed fat-associated differences before or after intervention |

Fat intake was categorized as ≤30% or >30% of total daily energy intake. Candidate genera were selected from Random Forest importance plots within each intake group. Paired pre–post comparisons (Wilcoxon signed-rank test) showed no significant changes in *Paraprevotella* or *Alistipes* in the low-fat group, and no distinct taxa were identified in the high-fat group.

Supplementary Table 6. FDR-adjusted p-values for gut microbial genera with the highest discriminatory contribution to baseline microbiota differences between participants with and without clinical improvement. Only the top 3–4 genera based on mean decrease accuracy were tested.

| Outcome | Genus | p-value | q-value (FDR) |
| --- | --- | --- | --- |
| Body weight improvement | ***Clostridium sensu stricto 1*** | **0.015** | **0.046** |
|  | *Faecalibacterium* | 0.157 | 0.236 |
|  | *Parasutterella* | 0.396 | 0.396 |
| ALT improvement | ***Faecalibacterium*** | **0.015** | **0.046** |
|  | *Granulicatella* | 0.254 | 0.370 |
|  | *Sutterella* | 0.370 | 0.370 |
| GGT improvement | ***Faecalibacterium*** | **0.014** | **0.042** |
|  | *[Eubacterium]_hallii_group* | 0.170 | 0.256 |
|  | *Veillonella* | 0.948 | 0.948 |
| Steatosis improvement in MRI | *Parasutterella* | 0.006 | 0.018 |
|  | ***Lachnospiraceae_ND3007_group*** | **0.009** | **0.018** |
|  | *Subdoligranulum* | 0.134 | 0.178 |
|  | *Faecalibacterium* | 0.460 | 0.460 |

*p*-values were calculated using the Mann–Whitney U test and adjusted for multiple comparisons using the Benjamini–Hochberg false discovery rate (FDR) method.


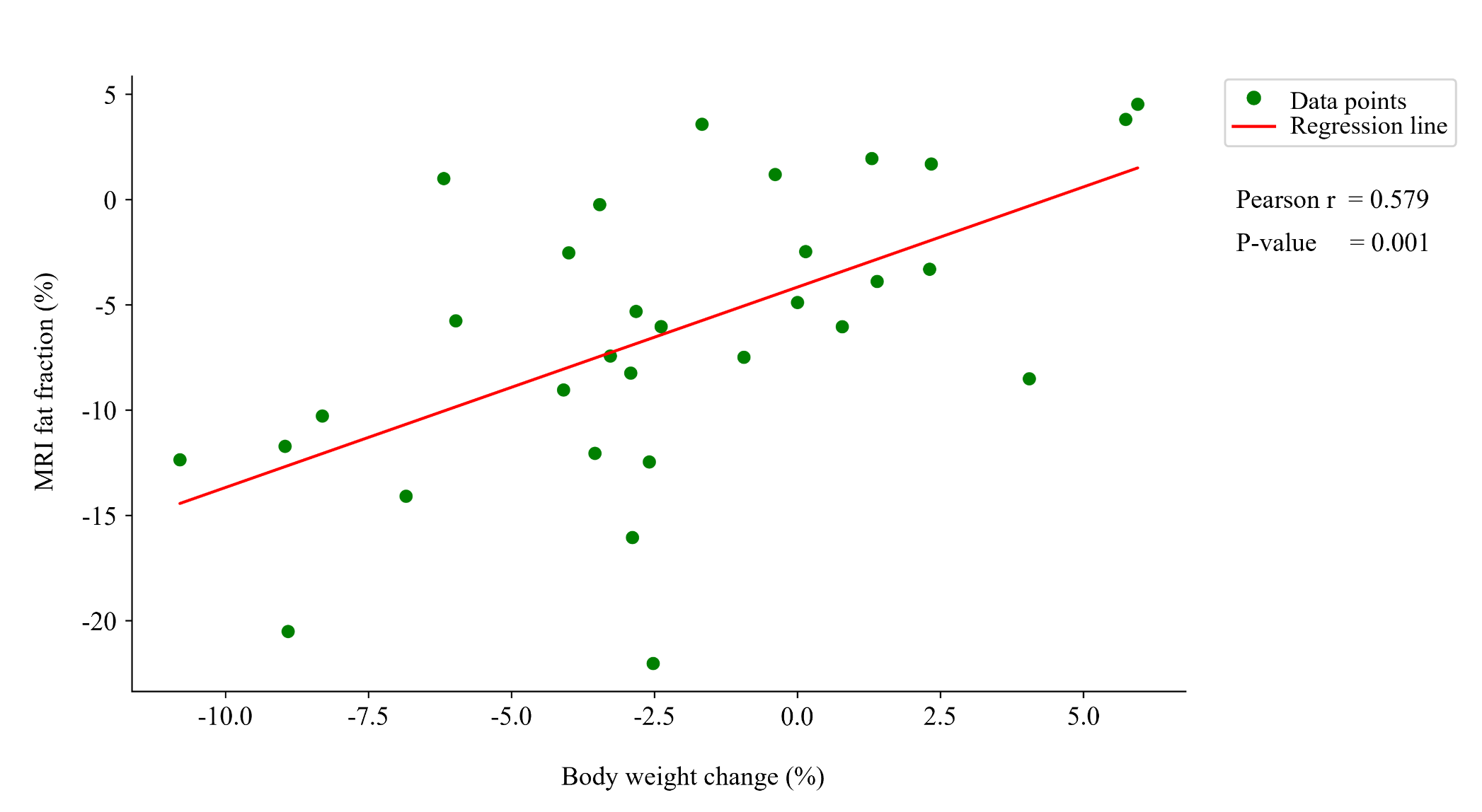


Supplementary Figure 1. Correlation between relative changes in body weight and MRI-measured hepatic fat fraction. Each dot represents an individual participant. Pearson’s r = 0.579, *p* = 0.001. Participants achieving ≥ 7% weight loss showed significantly greater hepatic fat reduction (*p* < 0.05).


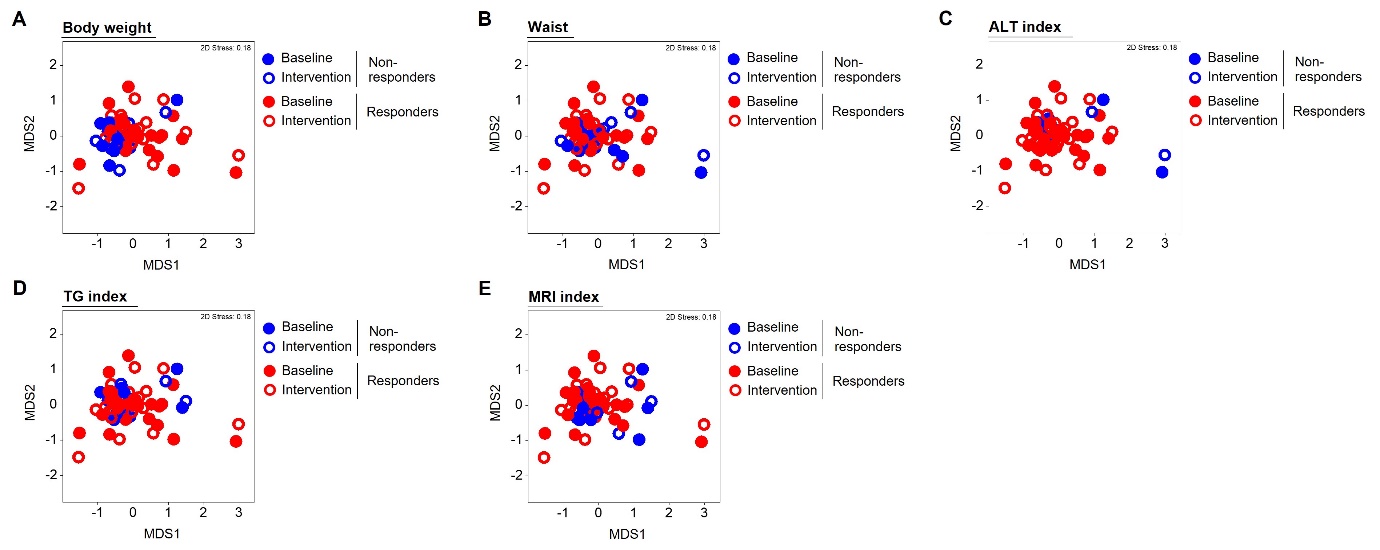


Supplementary Figure 2. Beta diversity (NMDS plot) of non-responders and responders about each clinical index before and after exercise-intervention. **a**. Body weight; **b**. Waist circumference; **c**. Alanine aminotransaminase; **d**. Triglycerides; **e**. MRI fat fraction. *ALT, alanine aminotransaminase*; *TG, tryglyceride*.


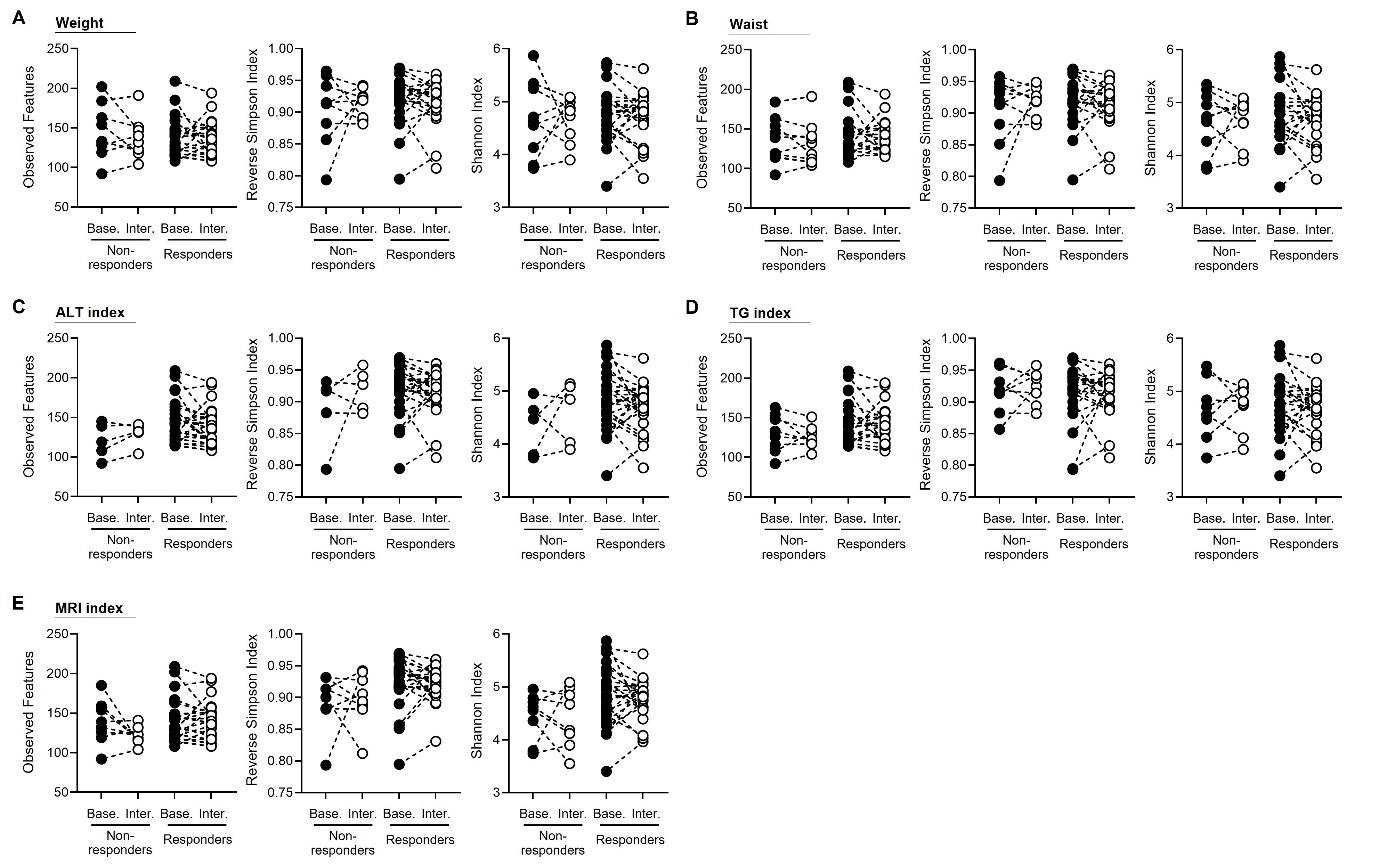


Supplementary Figure 3. Alpha diversity (observed Features, Simpson index, and Shannon index) of non-responders and responders about each clinical index before and after exercise-intervention. **a**. Body weight; **b**. Waist circumference; **c**. Alanine aminotransaminase; **d**. Triglycerides; **e**. MRI fat fraction. *ALT, alanine aminotransaminase*; *TG, tryglyceride*.


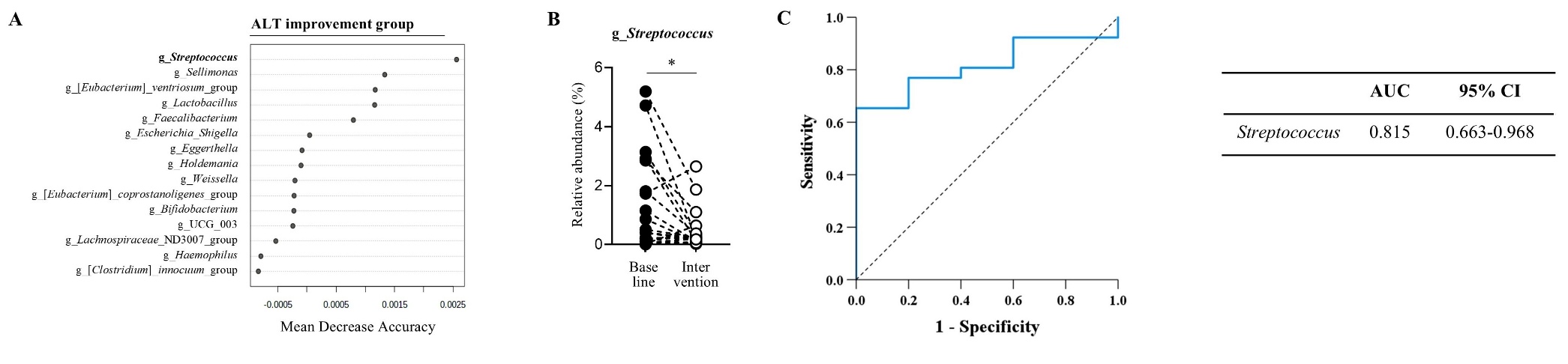
 Supplementary Figure 4. Gut microbial genera exhibiting a significant difference in the abundance before and after exercise-intervention. a. Random Forest analysis in ALT improvement group; b. *Streptococcus* abundance in ALT improvement group; c. ROC curve for each genus; ALT, alanine aminotransaminase; Wilcoxon matched-pairs rank tests was performed. *P < 0.05, **P < 0.01.
