# Supplementary material for: Clinical and microbial correlates of response to lifestyle intervention in pediatric metabolic dysfunction-associated steatotic liver disease
Source: Gut Pathog. 2026 Jan 18;18:6. doi: 10.1186/s13099-026-00798-5 (PMC12831411; doi:10.1186/s13099-026-00798-5)
Supplement: Supplementary file 2 — Supplementary Material 2. [file 13099_2026_798_MOESM2_ESM.docx]

The Protocol of HEALKIDS

**Clinical and Microbial correlates of response to lifestyle intervention in pediatric metabolic dysfunction-associated steatotic liver disease**

**Study Principal Investigator:** Jae Sung Ko, MD, PhD

Affiliation: Department of Pediatrics, Seoul National University College of Medicine

Address: 101 Daehak-ro, Jongno-Gu, Seoul, 03080, Korea

Email address: [kojs@snu.ac.kr](mailto:kojs@snu.ac.kr)

**Study Co-investigators:**

Jong Woo Hahn MD, PhD, Seoul National University College of Medicine

Jin Soo Moon MD, PhD, Seoul National University College of Medicine

Seunghyun Lee MD, PhD, Seoul National University College of Medicine

Donghyun Kim, PhD, Seoul National University College of Medicine

Contents

1. Background 1

2. Objectives 3

3. Study design 4

4. Participants 4

5. Sample size determination 5

6. Intervention 6

7. Measurement 11

8. Data analysis 14

9. Ethical considerations 14

10. Financing 15

11. References 16

**1. Background**

Non-Alcoholic Fatty Liver Disease (NAFLD) is diagnosed when fat accumulation in the liver exceeds 5% and is classified into non-alcoholic fatty liver (NAFL) and non-alcoholic steatohepatitis (NASH) based on pathological findings. With advancements in imaging technology, a meta-analysis published in the UK in 2021 reported that magnetic resonance imaging (MRI) could be used to diagnose NAFLD and assess its severity^1^. The prevalence of NAFLD has been steadily increasing, with a global prevalence of 25.2% in 2016 and a prevalence of 9.6% among children^2,3^. In South Korea, studies have reported a prevalence of 30.3% among adults, and it is becoming one of the most common chronic liver diseases in children as well^4^. The primary causes of NAFLD include high-calorie diets, excessive fat intake, and carbohydrate-rich Western-style diets, along with obesity. Additionally, genetic factors such as PNPLA3 and TM6SF have been identified^5^. Approximately 20% of NAFL patients progress to NASH, and 20% of NASH cases further develop into liver cirrhosis, potentially leading to liver failure and necessitating liver transplantation^6^. Given the heterogeneity of the disease, the term "Metabolic Dysfunction-Associated Fatty Liver Disease (MAFLD)" has been introduced to better reflect its association with obesity, diabetes, and metabolic syndrome^7^.

Lifestyle modifications, including diet and exercise, are considered the most effective treatment for NAFLD^5^. Several randomized controlled trials have demonstrated the benefits of exercise in improving NAFLD outcomes. A 2018 study conducted in Hong Kong involving 154 NAFLD patients assessed liver fat improvement through MRI after 12 months of dietary and exercise interventions^8^. Similarly, a 2020 study in Ireland with 24 NAFLD patients reported significant histological liver improvements after 12 weeks of aerobic exercise^9^.

Research on gut microbiota was initially conducted in obesity and type 2 diabetes using fecal microbiota transplantation in mice and has since been extended to NAFLD pathogenesis. Changes in gut microbiota due to high-fat diets and obesity lead to decreased microbial gene diversity and altered intestinal barrier function. This results in decreased short-chain fatty acids and increased lipopolysaccharides, which lower oxidative stress resistance and increase insulin resistance, thereby contributing to NAFLD progression^10,11^. Studies have shown increased gut permeability in NAFLD patients compared to controls, promoting inflammation and liver fat accumulation^12^. Furthermore, a study in the United States reported reduced alpha diversity in the gut microbiota of NAFLD patients^13^, while research in France demonstrated that gut microbiota composition changes with NAFLD severity^14^. Additionally, a study in the United States observed that gut viral diversity decreases as NAFLD severity increases^15^.

Several clinical interventions have been explored to modify gut microbiota composition and influence NAFLD progression. These include the administration of prebiotics^16^, synbiotics^17^, antibiotics^18^, dietary adjustments^19^, and exercise^20,21^. A 2019 German study with 44 NAFLD patients examined the effects of an 8-week web-based exercise program, reporting improvements in liver enzyme levels, increased microbial richness, and changes in gut microbiota composition. Specifically, Bacteroidetes and Euryarchaeota increased, while Actinobacteria decreased. Additionally, markers of steatosis and fibrosis, such as the fatty liver index and APRI (AST to platelet ratio index), were reduced^21^. Another study in the United States in 2021 with 6 NASH patients demonstrated reductions in body weight, liver enzyme levels, and MRI-measured liver fat fraction following a 20-week exercise intervention^20^.

Research on bariatric surgery has provided insights into the timeframe required for gut microbiota changes. Studies conducted in France (2010) and the United States (2013) reported significant shifts in microbial composition three months post-surgery^22,23^.

With the rising global prevalence of obesity and NAFLD, lifestyle modifications remain the primary treatment approach. However, there is no standardized dietary or exercise program for NAFLD management, particularly for children, who face additional challenges due to school and extracurricular schedules. Lessons can be drawn from structured exercise programs in other disease populations. A 2019 Dutch study found that a virtual reality-based aerobic exercise program improved Parkinson’s disease severity^24^. A 2021 Australian study demonstrated that an app-based exercise program reduced fall rates in older adults^25^. Additionally, a 2019 German study reported reductions in weight and body fat following an 8-week structured web-based exercise program for NAFLD patients^26^. Another German study in 2021 investigated the effects of web-based exercise programs in pediatric congenital heart disease patients^27^.

To quantitatively assess physical activity, the Metabolic Equivalent of Task (MET) system has been used in various studies. This system defines 1 MET as the energy expenditure of 1 kcal per kg per hour, allowing for the standardization of different exercise intensities^28,29^.

**2. Objectives**

The COVID-19 pandemic has led to increased consumption of delivery food and reduced outdoor activity, exacerbating the obesity epidemic and related metabolic diseases. While lifestyle modifications are crucial for NAFLD treatment, no standardized programs exist to encourage exercise. Furthermore, there are no established biomarkers in blood or stool that can accurately predict NAFLD severity or prognosis. Therefore, further research is needed to analyze the impact of lifestyle modifications on NAFLD and explore gut microbiome-based biomarkers for disease severity and prognosis.

**3. Study design and process**

**3.1 Study design**

This study is a nonrandomized clinical trial, prospective, single–arm intervention in liver fat, liver function, and the gut microbiome following 12 weeks of lifestyle modifications, including diet and exercise.

**4. Participants**

**4.1 Inclusion criteria**

Pediatric patients aged 10 years and older but under 19 years who visited Seoul National University Children's Hospital and were diagnosed with hepatic steatosis on liver MRI and satisfied at least one cardiometabolic criterion, while having no other identifiable cause of steatosis.

**4.2 Exclusion criteria**

1. Patients with other liver diseases (including viral hepatitis [HBV, HCV])

2. Autoimmune hepatitis, metabolic liver diseases, or muscular diseases

3. Those who had taken antibiotics within the past 3 months

4. Patients who could not discontinue probiotics

5. Individuals with difficulties in cooperation for testing

6. Parents or legal guardians who had difficulty understanding the study explanation

7. Patients for whom MRI imaging was contraindicated

8. Other cases where participation in the study was deemed inappropriate

**5. Sample size determination**

A study conducted by Wong compared the degree of hepatic fat improvement in participants with a BMI over 25 using MRI in both the intervention and control groups. The results indicated hepatic fat improvement rates of 61% and 21% in the intervention and control groups, respectively^8^. Based on this, calculating the required sample size using a desired power of 0.8, a significance level of 0.05 (two-sided), an anticipated known population improvement of 21%, and an expected study group improvement of 61% resulted in a sample size of 9 participants. Additionally, when comparing improvements in liver enzyme levels between the intervention and control groups, the rates were found to be 49% and 23%, respectively. Using the same calculation parameters with an anticipated known population improvement of 23% and a study group improvement of 49%, the required sample size was determined to be 23 participants. Considering multiple outcome variables and aiming to increase the precision of the estimates while accounting for a dropout rate of 10–15%, the final sample size was set at 40 participants.

**6. Intervention**

**6.1 Overall intervention program**

Participants were encouraged to engage in aerobic physical activity for more than 1 hour at least 5 times a week, with moderate or higher intensity. They aimed to achieve a target of 10,000 steps per day using the provided wearable devices to monitor their weekly step count. Additionally, they were instructed to complete the International Physical Activity Questionnaire weekly, recalling the number of days and hours of physical activity, walking time, and sedentary time over the past week, and submit it during outpatient visits. Regarding the wearable devices, participants recorded the number of days worn and the average daily wear time each week. For the analysis of physical activity, participants reported their daily step count on a weekly basis and were classified as follows: those with fewer than 5,000 steps were categorized as the sedentary group, those with 5,000 to 7,500 steps as the low activity group, those with 7,500 to 10,000 steps as the moderate activity group, and those with over 10,000 steps as the high activity group.^30^ Through the survey, participants were classified based on MET-minutes per week as follows: those with less than 600 MET-minutes per week were categorized as the low activity group, those with 600 to 3,000 MET-minutes as the moderate activity group, and those with over 3,000 MET-minutes as the high activity group.^31^ All study participants received dietary evaluations and appropriate dietary education from a nutritionist at the beginning of the study and at 4, 8, and 12 weeks. They were instructed to reduce their overall caloric intake, particularly their intake of sugar and fat. Participants were encouraged to keep a food diary to record their total daily intake of carbohydrates, protein, fat, and total sugar consumption at each outpatient visit. The food diary prompted participants to provide detailed records of the foods, snacks, and beverages consumed on two weekdays and one weekend day, including the time, location, food name, and quantity consumed. Dietary compliance was evaluated based on total average daily intake, the proportion of carbohydrates, protein, fat, and total sugar intake. Total average daily intake was adjusted based on the patient’s height and activity level, and participants were classified into groups that consumed less or more than the adjusted daily recommended intake. Carbohydrate intake was classified based on a standard of 55% of total intake, protein based on 20%, and fat based on 30%.^32^ Total sugar intake was categorized into low and high groups based on the median intake of the participants. To enhance compliance, social networking service was utilized to send daily messages encouraging dietary control and exercise. Weekly reminders were sent to ensure participants did not miss filling out their food diaries and exercise questionnaires, and inquiries regarding diet or exercise were addressed throughout the study.

**7. Measurement**

**7.1 Clinical assessments**

At the beginning and end of the study, Z scores were calculated for weight, height, body mass index (BMI), blood pressure, and waist circumference according to sex and age. Additionally, blood tests were conducted to measure alanine aminotransaminase (ALT), aspartate aminotransferase (AST), alkaline phosphatase (ALP), and γ-glutamyl transferase (GGT), as well as fasting glucose, fasting insulin, total cholesterol, HDL-Cholesterol, LDL-Cholesterol, Triglycerides, and HbA1c. Based on these measurements, calculations were performed for HOMA-IR (Homeostatic Model Assessment for Insulin Resistance), APRI (AST to Platelet Ratio Index), fatty liver index (FLI), and hepatic steatosis index (HSI). HOMA-IR was calculated using the following formula: HOMA-IR= fasting insulin (µU/ml) × fasting plasma glucose (mg/dl)/405. APRI was calculated as (AST/upper limit of normal)/platelet count (x10^9^/L) x 100. FLI was calculated using the following formula: FLI= (e^0.953 * loge (triglycerides) + 0.139 * BMI+0.718 * loge (ggt) + 0.053 * waist circumference - 15.745^) / (1 + e^0.953 * loge (triglycerides) + 0.139 * BMI+0.718 * loge (ggt) + 0.053 * waist circumference- 15.745^) * 100. HSI was calculated using the following formula: HSI= 8 * (ALT/AST) + BMI + 2 (if type 2 diabetes) + 2 (if female).

**7.2 Liver fat measurement**

At the beginning and end stages of the study, the degree of steatosis is assessed using liver MRI as follows: grade 0 (healthy, <6.4%), grade 1 (mild, 6.4%-17.4%), grade 2 (moderate, 17.4%-22.1%), and grade 3 (severe, >22.1%).^33^ Additionally, the extent of fibrosis is evaluated as follows: no fibrosis (F0, <2.5 kPa), mild fibrosis (F1, 2.5–3.0 kPa), moderate fibrosis (F2, 3.0–3.5 kPa), severe fibrosis (F3, 3.5–4.0 kPa), or cirrhosis (F4, 4.0–4.5 kPa).^34^ The MRI examination typically takes around 20 minutes and utilizes a pediatric liver disease MRI scan protocol using the GE Healthcare Premier 3T MRI.

Liver MRI scan protocol includes chemical shift-encoded MRI proton-density fat fraction (PDFF) and MR elastography (MRE). Chemical shift–encoded MRI PDFF estimation was the iterative decomposition of water and fat using echo-asymmetry and the least-squares estimation quantitative sequence (IDEAL IQ; GE Healthcare) to estimate the PDFF map in the axial plane. One pediatric radiologist (S.L. with 12 years of experience in pediatric liver MRI) manually placed a 1-cm-diameter circular region of interest in each of the right hepatic lobes of the PDFF map.^33^ Averaged values from liver segments five through eight were calculated and used as the reference hepatic fat content value.^35^ As previously proposed, the degree of hepatic steatosis was graded as mild (S1), moderate (S2), and severe (S3) at MRI PDFF thresholds of 6.4%, 17.4%, and 22.1%, respectively.^33,36^

MRE images were obtained with the patients placed in the supine position with a passive driver attached to the right anterior chest wall. Axial slices containing the largest volume of the liver parenchyma were acquired using the spin-echo MRE technique with a single breath-hold during end-expiration. Liver stiffness measurement was performed by drawing freehand ROIs on the stiffness maps, only on valid areas of 95% confidence maps to include the largest part of the liver parenchyma of the right lobe), while excluding vessels and liver edges.^37^ The arithmetic means of the measured stiffness values (in kPa) from all available ROIs (maximum of four ROIs in each subject) was determined as the representative liver stiffness of a subject.

**7.3 Fecal microbiota analysis**

Stool samples are collected in specimen containers, with each containing 5g of stool, and then stored at -70°C in a freezer after preprocessing. Microbial gene profiling of the gut microbiota is conducted through 16S rRNA amplicon sequencing from the stool samples. Following the 12-week exercise intervention, the analysis of gut microbiota is repeated to compare the changes in the gut microbiota of the participant groups. Briefly, total bacterial DNA was extracted using E.Z.N.A.® Stool DNA Kit (Omega Bio-Tek) according to the manufacturer’s instructions with an addition of extensive bead-beating lysis step. The V3-V4 regions of the bacterial 16S rRNA were amplified using 341-F (5’-CCTACGGGNGGCWGCAG-3’) /805-R (5’-GACTACHVGGGTATCTAATCC-3’) primer set (Cosmogenetech) and OneTaq Hot Start 2X Master Mix (New England Biolabs). Amplicons were quantified by GenNext NGS Library Quantification Kit (Toyobo) according to the manufacturer’s instructions and sequencing was performed on an Illumina MiSeq system using MiSeq Reagent Kit v2 (500-cycles). Raw sequence data were processed with QIIME 2 (version 2020.08). Taxonomic assignment against the SILVA database ver. 132 was performed using the integrated analysis tools within QIIME 2. Observed features were employed as an alpha-diversity, which estimates microbial richness based on the number of unique bacterial taxa observed in a sample. Also, Simpson and Shannon indices, which captures both the richness and the evenness of the bacterial taxa present, was quantified as another alpha-diversity parameter. Non-metric multidimensional scaling (NMDS) plots, which intuitively show the major bacterial compositional differences between each sample, were generated and visualized by Primer 7 software (PRIMER-e, New Zealand). Moreover, we identified candidate bacterial genera or feature taxa using the Random Forest analysis in MicrobiomeAnalyst 2.0 (<https://new.microbiomeanalyst.ca/>).

**8. Data analysis**

Continuous variables were expressed as mean and standard deviation or median and interquartile range, and compared using independent t-tests or Mann-Whitney U tests. Categorical variables were compared using the Chi-square test. Paired t-tests or Wilcoxon signed-rank tests were used to assess within-group differences in data before and after the study. The impact of dietary control and physical activity changes on biochemical parameters and liver steatosis was evaluated using multiple regression analysis, selecting explanatory variables for model construction using the stepwise method. A *p*-value of less than 0.05 was considered statistically significant. Gut microbiome analysis assessed alpha diversity and beta diversity. SPSS (IBM Corp Released 2017, IBM SPSS Statistics for Windows, Version 25; IBM Corp, Armonk, New York) software was used to perform statistical analysis.

**9. Ethical considerations**

**9.1 Benefits and Risk of study participants**

Participants in this study will benefit from a more detailed assessment of liver fat levels and fibrosis improvement. However, investigators will inform the participants' legal guardians that the data obtained through these tests cannot be immediately used for diagnosis or treatment based on current medical knowledge. Additionally, since there is no established standard for interpreting the results as favorable or unfavorable, and the report content may be difficult for the general public to understand, these aspects will be explained during the consent process. Although blood tests are invasive, they do not pose a significant risk of serious complications and will not be conducted beyond the scope of routine clinical care. Imaging tests involve the use of contrast agents but are generally considered safe. Stool samples will be collected from participants' routine bowel movements, posing no additional risk to the participants.

**9.2 Approval of Institutional review board**

This study was approved by the Institutional Review Board (No. 2207-177-1345) at Seoul National University Hospital, Korea, and all methods were performed in accordance with the relevant guidelines and regulations.

**9.3 Registration of clinical trial**

The Clinical Research Information Service of the Korea Center for Disease Control and Prevention Identifier: KCT0010340

**10. Financing**

Research reported in this publication was supported by National Research Foundation of Korea (2022R1F1A1071396 and RS-2023-00227939).

**11. References**

1. Selvaraj EA, Mozes FE, Jayaswal ANA, et al. Diagnostic accuracy of elastography and magnetic resonance imaging in patients with NAFLD: A systematic review and meta-analysis. J Hepatol 2021;75:770-785.

2. Younossi ZM, Koenig AB, Abdelatif D, Fazel Y, Henry L, Wymer M. Global epidemiology of nonalcoholic fatty liver disease-Meta-analytic assessment of prevalence, incidence, and outcomes. Hepatology 2016;64:73-84.

3. Yu EL, Schwimmer JB. Epidemiology of Pediatric Nonalcoholic Fatty Liver Disease. Clin Liver Dis (Hoboken) 2021;17:196-199.

4. Im HJ, Ahn YC, Wang JH, Lee MM, Son CG. Systematic review on the prevalence of nonalcoholic fatty liver disease in South Korea. Clin Res Hepatol Gastroenterol 2021;45:101526.

5. European Association for the Study of the L, European Association for the Study of D, European Association for the Study of O. EASL-EASD-EASO Clinical Practice Guidelines for the management of non-alcoholic fatty liver disease. J Hepatol 2016;64:1388-1402.

6. Sheka AC, Adeyi O, Thompson J, Hameed B, Crawford PA, Ikramuddin S. Nonalcoholic Steatohepatitis: A Review. JAMA 2020;323:1175-1183.

7. Eslam M, Alkhouri N, Vajro P, et al. Defining paediatric metabolic (dysfunction)-associated fatty liver disease: an international expert consensus statement. Lancet Gastroenterol Hepatol 2021;6:864-873.

8. Wong VW, Wong GL, Chan RS, et al. Beneficial effects of lifestyle intervention in non-obese patients with non-alcoholic fatty liver disease. J Hepatol 2018;69:1349-1356.

9. O'Gorman P, Naimimohasses S, Monaghan A, et al. Improvement in histological endpoints of MAFLD following a 12-week aerobic exercise intervention. Aliment Pharmacol Ther 2020;52:1387-1398.

10. Aron-Wisnewsky J, Warmbrunn MV, Nieuwdorp M, Clement K. Nonalcoholic Fatty Liver Disease: Modulating Gut Microbiota to Improve Severity? Gastroenterology 2020;158:1881-1898.

11. Tilg H, Cani PD, Mayer EA. Gut microbiome and liver diseases. Gut 2016;65:2035-2044.

12. Luther J, Garber JJ, Khalili H, et al. Hepatic Injury in Nonalcoholic Steatohepatitis Contributes to Altered Intestinal Permeability. Cell Mol Gastroenterol Hepatol 2015;1:222-232.

13. Schwimmer JB, Johnson JS, Angeles JE, et al. Microbiome Signatures Associated With Steatohepatitis and Moderate to Severe Fibrosis in Children With Nonalcoholic Fatty Liver Disease. Gastroenterology 2019;157:1109-1122.

14. Boursier J, Mueller O, Barret M, et al. The severity of nonalcoholic fatty liver disease is associated with gut dysbiosis and shift in the metabolic function of the gut microbiota. Hepatology 2016;63:764-775.

15. Lang S, Demir M, Martin A, et al. Intestinal Virome Signature Associated With Severity of Nonalcoholic Fatty Liver Disease. Gastroenterology 2020;159:1839-1852.

16. Bomhof MR, Parnell JA, Ramay HR, et al. Histological improvement of non-alcoholic steatohepatitis with a prebiotic: a pilot clinical trial. Eur J Nutr 2019;58:1735-1745.

17. Scorletti E, Afolabi PR, Miles EA, et al. Synbiotics Alter Fecal Microbiomes, But Not Liver Fat or Fibrosis, in a Randomized Trial of Patients With Nonalcoholic Fatty Liver Disease. Gastroenterology 2020;158:1597-1610 e1597.

18. Chong CYL, Orr D, Plank LD, Vatanen T, O'Sullivan JM, Murphy R. Randomised Double-Blind Placebo-Controlled Trial of Inulin with Metronidazole in Non-Alcoholic Fatty Liver Disease (NAFLD). Nutrients 2020;12.

19. Ghetti FF, De Oliveira DG, De Oliveira JM, Ferreira L, Cesar DE, Moreira APB. Effects of Dietary Intervention on Gut Microbiota and Metabolic-Nutritional Profile of Outpatients with Non-Alcoholic Steatohepatitis: a Randomized Clinical Trial. J Gastrointestin Liver Dis 2019;28:279-287.

20. Hughes A, Dahmus J, Rivas G, et al. Exercise Training Reverses Gut Dysbiosis in Patients With Biopsy-Proven Nonalcoholic Steatohepatitis: A Proof of Concept Study. Clin Gastroenterol Hepatol 2021;19:1723-1725.

21. Huber Y, Pfirrmann D, Gebhardt I, et al. Improvement of non-invasive markers of NAFLD from an individualised, web-based exercise program. Aliment Pharmacol Ther 2019;50:930-939.

22. Furet JP, Kong LC, Tap J, et al. Differential adaptation of human gut microbiota to bariatric surgery-induced weight loss: links with metabolic and low-grade inflammation markers. Diabetes 2010;59:3049-3057.

23. Kong LC, Tap J, Aron-Wisnewsky J, et al. Gut microbiota after gastric bypass in human obesity: increased richness and associations of bacterial genera with adipose tissue genes. Am J Clin Nutr 2013;98:16-24.

24. van der Kolk NM, de Vries NM, Kessels RPC, et al. Effectiveness of home-based and remotely supervised aerobic exercise in Parkinson's disease: a double-blind, randomised controlled trial. Lancet Neurol 2019;18:998-1008.

25. Delbaere K, Valenzuela T, Lord SR, et al. E-health StandingTall balance exercise for fall prevention in older people: results of a two year randomised controlled trial. BMJ 2021;373:n740.

26. Pfirrmann D, Huber Y, Schattenberg JM, Simon P. Web-Based Exercise as an Effective Complementary Treatment for Patients With Nonalcoholic Fatty Liver Disease: Intervention Study. J Med Internet Res 2019;21:e11250.

27. Meyer M, Brudy L, Fuertes-Moure A, et al. E-Health Exercise Intervention for Pediatric Patients with Congenital Heart Disease: A Randomized Controlled Trial. J Pediatr 2021;233:163-168.

28. Ainsworth BE, Haskell WL, Herrmann SD, et al. 2011 Compendium of Physical Activities: a second update of codes and MET values. Med Sci Sports Exerc 2011;43:1575-1581.

29. Mendes MA, da Silva I, Ramires V, et al. Metabolic equivalent of task (METs) thresholds as an indicator of physical activity intensity. PLoS One 2018;13:e0200701.

30. Darvall JN, Wang A, Nazeem MN, Harrison CL, Clarke L, Mendoza C, et al. A Pedometer-Guided Physical Activity Intervention for Obese Pregnant Women (the Fit MUM Study): Randomized Feasibility Study. JMIR Mhealth Uhealth. 2020;8(5):e15112.

31. Lear SA, Hu W, Rangarajan S, Gasevic D, Leong D, Iqbal R, et al. The effect of physical activity on mortality and cardiovascular disease in 130 000 people from 17 high-income, middle-income, and low-income countries: the PURE study. Lancet. 2017;390(10113):2643-54.

32. Trumbo P, Schlicker S, Yates AA, Poos M, Food, Nutrition Board of the Institute of Medicine TNA. Dietary reference intakes for energy, carbohydrate, fiber, fat, fatty acids, cholesterol, protein and amino acids. J Am Diet Assoc. 2002;102(11):1621-30.

33. Tang A TJ, Sun M, Hamilton G, Bydder M, Wolfson T, Gamst AC, Middleton M, Brunt EM, Loomba R, Lavine JE, Schwimmer JB, Sirlin CB. Nonalcoholic Fatty Liver disease: MR imaging of liver proton density fat fraction to assess hepatic steatosis. Radiology. 2013;267(2):422-31.

34. Hoodeshenas S, Yin M, Venkatesh SK. Magnetic Resonance Elastography of Liver: Current Update. Top Magn Reson Imaging. 2018;27(5):319-33.

35. Jung J, Han A, Madamba E, Bettencourt R, Loomba RR, Boehringer AS, et al. Direct Comparison of Quantitative US versus Controlled Attenuation Parameter for Liver Fat Assessment Using MRI Proton Density Fat Fraction as the Reference Standard in Patients Suspected of Having NAFLD. Radiology. 2022;304(1):75-82.

36. Middleton MS, Van Natta ML, Heba ER, Alazraki A, Trout AT, Masand P, et al. Diagnostic accuracy of magnetic resonance imaging hepatic proton density fat fraction in pediatric nonalcoholic fatty liver disease. Hepatology. 2018;67(3):858-72.

37. Chang W LJ, Yoon JH, Han JK, Choi BI, Yoon JH, Lee KB, Lee KW, Yi NJ, Suh KS. Liver Fibrosis Staging with MR Elastography: Comparison of Diagnostic Performance between Patients with Chronic Hepatitis B and Those with Other Etiologic Causes. Radiology. 2016;280(1):88-97.
